# Significance of the board-certified surgeon systems and clinical practice guideline adherence to surgical treatment of esophageal cancer in Japan: a questionnaire survey of departments registered in the National Clinical Database

**DOI:** 10.1007/s10388-019-00672-1

**Published:** 2019-04-12

**Authors:** Yasushi Toh, Hiroyuki Yamamoto, Hiroaki Miyata, Mitsukazu Gotoh, Masayuki Watanabe, Hisahiro Matsubara, Yoshihiro Kakeji, Yasuyuki Seto

**Affiliations:** 1 The Japan Esophageal Society, Tokyo, Japan; 2 National Clinical Database, Tokyo, Japan; 3 The Japanese Society of Gastroenterological Surgery, 3-1-17 Axior Mita 6F, Minato-ku, Tokyo, 108-0073 Japan

**Keywords:** Esophageal cancer, Esophagectomy, Quality indicator, Board-certified surgeon, Questionnaire survey

## Abstract

**Background:**

It remains unknown how much institutional medical structure and process of implementation of clinical practice guidelines for esophageal cancers can improve quality of surgical outcome in Japan.

**Methods:**

A web-based questionnaire survey was performed for departments registered in the National Clinical Database in Japan from October 2014 to January 2015. Quality indicators (QIs) including structure and process indicators (clinical practice guideline adherence) were evaluated on the risk-adjusted odds ratio for operative mortality (AOR) of the patients using registered cases in the database who underwent esophagectomy and reconstruction in 2013 and 2014.

**Results:**

Among 916 departments which registered at least one esophagectomy case during the study period, 454 departments (49.6%) responded to the questionnaire. Analyses of 6661 cases revealed that two structure QIs (certification of training hospitals by Japan Esophageal Society and presence of board-certified esophageal surgeons) were associated with significantly lower AOR (*p* < 0.001 and *p* = 0.005, respectively). One highly recommended process QI regarding preoperative chemotherapy had strong tendency to associate with lower AOR (*p* = 0.053). In two process QIs, the answer “performed at the doctor’s discretion” showed a significant negative impact on prognosis, suggesting importance of institutional uniformity.

**Conclusions:**

The medical institutional structure of board-certified training sites for esophageal surgeons and of participation of board-certified esophageal surgeons improves surgical outcome in Japan. Establishment of appropriate QIs and their uniform implementation would be crucial for future quality improvement of medical care in esophagectomy.

## Introduction

Quality of care is measured by “the degree to which health services for individuals and populations increase the likelihood of the desired health outcomes and are consistent with current professional knowledge” (Institute of Medicine: http://www.nationalacademies.org/hmd/). In recent years, while many clinical practice guidelines have been developed for the management of cancer in Japan, the evidence–practice gap, i.e., incomplete utilization or dissemination of current clinical knowledge in clinical practice, remains a problem [1, 2]. The first measure to resolve this problem is to try to understand the medical care system, such as the appropriate distribution of health professionals and equipment in clinical settings, and the status of implementation of clinical practice guidelines for various cancers (evaluation of structure and process in the Donabedian model [3]) using established quality indicators (QIs) throughout Japan and to compare them with indicators of the level of medical care, such as the incidence of complications, treatment-related mortality, and survival rate. Differences in the results between each institution and the whole country are required to be fed back to the relevant institution as individual treatment outcomes to make the quality of medical care uniform across institutions in Japan, and for future reconsideration of clinical practice guidelines and standards of care.

The National Clinical Database (NCD) is a registry of surgical cases established in 2010 by 10 academic societies that are affiliated to the board-certified surgeon system [4], including the Japan Surgical Society (JSS) and the Japanese Society of Gastroenterological Surgery (JSGS) and consists of a network of more than 5,000 participating institutions and more than 1,02,000 departments throughout Japan as of August 2018. More than 95% of cases of surgeries performed in Japan are registered in the NCD [5]. In the field of gastroenterological surgery, detailed data on 8 major surgical procedures have been collected to prepare a risk model for each surgical procedure, which is utilized as a risk calculator for risk evaluations in clinical settings [6–13].

In cooperation with a study group, “a study on the utilization of high-accuracy organ cancer registration in clinical practice guidelines and medical specialist training,” of the Health and Labour Sciences Research Grant, the NCD conducted a questionnaire survey of the significance of board-certified surgeons in the treatment of various cancers (esophageal, gastric, colorectal, liver, pancreatic, biliary tract, lung and breast cancers) and the status of implementation of guidelines in each of the departments of institutions registered in the NCD in 2014. Although the results have been reported in part in Japanese [14], the influence of the surveyed items for each cancer on indicators of the level of medical care, such as the incidence of postoperative complications, treatment-related mortality, and survival rate, has not been analyzed in detail. In the present paper, we report the results exclusively related to esophageal cancer, which was among the first investigated.

## Materials and methods

### Questionnaire survey

A web questionnaire page was created on the NCD registration system, and the department responsible for esophageal surgery at each of the registered institutions was requested to answer the questionnaire. The survey period was from October 1, 2014 to January 31, 2015. Of all, 454 of total 916 departments (49.6%) which registered at least one esophagectomy case in this period responded to the questionnaire. A total of 11,809 patients who underwent esophagectomy and reconstruction during this period in Japan were included in this study. The questionnaire items related to the treatment of esophageal cancer as QIs are shown in Table 1.

**Table 1:** The questionnaire items related to the treatment of esophageal cancer

Q1	Is your institution accredited by or related to the Japan Surgical Society (JSS)?
Q2	Is your institution certified by the Japanese Society of Gastroenterological Surgery (JSGS)?
Q3	Is your institution certified by the Japan Esophageal Society (JES)?
Q4	Is there a board-certified gastroenterological surgeon by JSGS?
Q5	Is there a board-certified esophageal surgeon by JES?
Q6	Is there a board-certified esophagologist by JES?
Q7	Do you screen for synchronous head and neck cancer in new patients with esophageal cancer?
Q8	Do you administer steroids in the perioperative period to patients scheduled to undergo esophagectomy and reconstruction for esophageal cancer?
Q9	Do you perform lymph node dissection around the bilateral recurrent laryngeal nerves (#101R, L, #106recR, L: [25, 26]) during resection and reconstruction for thoracic esophageal cancer?
Q10	Do you administer neoadjuvant chemotherapy for resectable stage II/III (T1-3N0/1M0, Union for International Cancer Control [UICC] classification, 2002 edition) thoracic esophageal cancer?
Q11	Do you measure the serum level of carcinoembryonic antigen (CEA) or squamous cell carcinoma antigen (SCC) (or both) during follow-up after esophagectomy?

For questions (Q) 7–11, the respondents selected one of three responses: not performed in principle (group A), performed in principle (group B), and recommended by the institution, but performed at the doctor’s discretion (group C).

Q7–Q11 were selected through discussion among council members of the Japan Esophageal Society (JES), mainly from those with higher recommendation grades in the 2012 Guidelines for Diagnosis and Treatment of Carcinoma of the Esophagus [15]. Actually, with regard to the medical care for esophageal cancer in Japan, it is often difficult to determine the grade of recommendation based on high-level evidence. Accordingly, the grades of recommendation were B (there is scientific evidence and it is recommended) for Q7–Q10 and C1 (there is no scientific evidence, but it is recommended) for Q11, and no questions with grade A recommendation (there is strong scientific evidence and it is strongly recommended), which was considered appropriate for the QIs, were selected.

### Relationships between the above QIs and the level of medical care

**Fig. 1:**
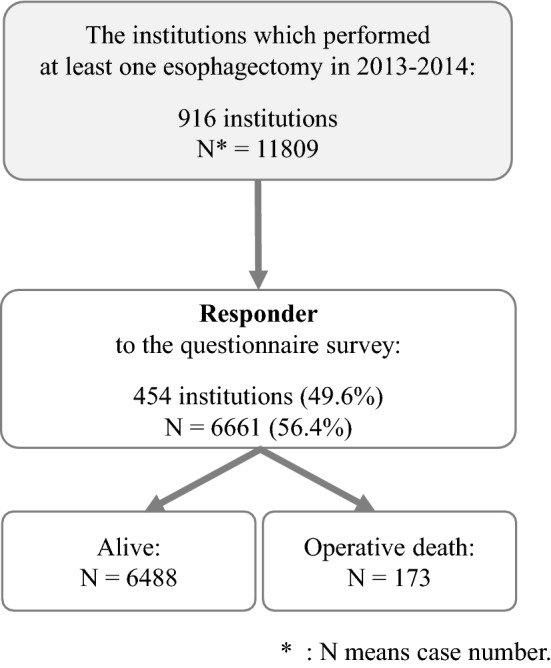
The flow of patient selection

Of the patients who underwent esophagectomy and reconstruction in 2013 or 2014 at the 454 departments that responded to the questionnaire, only those who did not refuse registration, had no missing data on their gender or outcome, and gave approval for participating in the survey were analyzed. Eventually, 6,661 (56.4%) patients were analyzed (the number of patients before examining the questionnaire data: 11,809 patients) (Fig. 1). Association between the QIs and operative mortality was evaluated for these patients. Here, the operative mortality was defined as death within the index hospitalization period regardless of the length of hospital stay (up to 90 days), as well as any death after discharge, up to 30 days after surgery. In esophagectomy, 90-day mortality is thought to be a better outcome measure because it provides a better understanding of true death risk for the surgeon and patient [16, 17].

### Multivariable regression analysis

To clarify the relationship between the answer of each questionnaires and operative mortality, multivariable logistic regression models fitted with generalized estimating equation were utilized, considering clustering of patients by hospital levels. To adjust patient-level risk factors, the following variables, which were risk model variables in the previous report [6], were utilized: age category, sex, preoperative activity of daily life (ADL) (any assistance), chronic obstructive pulmonary disease (COPD), cancer metastasis/relapse, weight loss, white blood cell (WBC) < 4500/μl, platelet < 120,000/μl, albumin < 3.5 g/dl, blood urea nitrogen (BUN) < 8 mg/dl, Serum Na < 138 mEq/l, and prothrombin time-international normalized ratio (PT-INR) > 1.25. The results were expressed as odds ratio (OR) and confidence interval (95% CI). Q1, Q4 and Q11 did not proceed to multivariable analyses, because distribution of the variables was extremely low (< 1%, Q1 and Q4) (Table 2) and guideline recommendation grade was low (Q11).

**Table 2:** The response distribution and relationship between each quality indicator and the crude operative mortality

Questionnaire item	Department No.	Operative death (*n* = 173)		Alive (*n* = 6488)		Total		Mortality rate
		Pt No.	%	Pt No.	%	No.	%	
Q1 Institution accredited by or related to JSS								*p* = 0.009*
No	4	2	1.2	11	0.2	13	0.2	15.38%
Accreditet	410	165	95.4	6377	98.3	6542	98.2	2.52%
Relater	40	6	3.5	100	1.5	106	1.6	5.66%
Q2 Institution certified by JSGS								*p* = 0.375*
Yes	389	166	96.0	6291	97.0	6457	96.9	2.57%
No	65	7	4.0	197	3.0	204	3.1	3.43%
Q3 Institution certified by JES								*p* < 0.001
Yes	98	81	46.8	4249	65.5	4330	65.0	1.87%
No	356	92	53.2	2239	34.5	2331	35.0	3.95%
Q4 Board-certified gastroenterological surgeon by JSGS								*p* = 1.000*
Yes	440	172	99.4	6437	99.2	6609	99.2	2.60%
No	14	1	0.6	51	0.8	52	0.8	1.92%
Q5 Board-certified esophageal surgeon by JES								*p* < 0.001
Yes	95	84	48.6	4115	63.4	4199	63.0	2.00%
No	359	89	51.4	2373	36.6	2462	37.0	3.61%
Q6 Board-certified esophagologist by JES								*p* = 0.001
Yes	159	112	64.7	4913	75.7	5025	75.4	2.23%
No	295	61	35.3	1575	24.3	1636	24.6	3.72%
Q7 Screen for synchronous head and neck cancer								*p* = 0.005*
Not performed in principle	39	5	2.9	307	4.7	312	4.7	1.60%
Performed in principle	325	145	83.8	5750	88.6	5895	88.5	2.46%
Doctor’s discretion^a^	90	23	13.3	431	6.6	454	6.8	5.07%
Q8 Administer steroids in the perioperative period								*p* < 0.001
Not performed in principle	215	63	36.4	2340	36.1	2403	36.1	2.62%
Performed in principle	178	87	50.3	3760	58.0	3847	57.8%	2.26%
Doctor’s discretion^a^	61	23	13.3	388	6.0	411	6.2	5.60%
Q9 Perform lymph node dissection around the bilateral recurrent laryngeal nerves								*p* = 0.001*
Not performed in principle	22	4	2.3	68	1.0	72	1.1	5.56%
Performed in principle	330	144	83.2	5962	91.9	6106	91.7	2.36%
Doctor’s discretion^a^	102	25	14.5	458	7.1	483	7.3	5.18%
Q10 Administer neoadjuvant chemotherapy for resectable stage II/III thoracic esophageal cancer								*p* = 0.029
Not performed in principle	53	18	10.4	379	5.8	397	6.0	4.53%
Performed in principle	308	133	76.9	5391	83.1	5524	82.9	2.41%
Doctor’s discretion^a^	93	22	12.7	718	11.1	740	11.1	2.97%

*JSS* Japan Surgical Society, *JSGS* Japanese Society of Gastroenterological Surgery (JSGS), *JES* Japan Esophageal Society

^a^Doctor’s discretion indicates “recommended by the institution, but performed at the doctor’s discretion”

*Fisher’s exact test

### Statistical analysis

Chi-square tests or Fisher’s exact tests were performed to compare categorical data and their distributions as appropriate. Two-sided probability values less than 0.05 are considered to be statistically significant. All statistical calculations were performed with STATA 15 (STATA Corp., TX, USA).

## Results

### The patient’s characteristics, the response distribution and the relationship between each result of QIs and the crude operative mortality

The patients’ characteristics in this study and their relationships with crude operative mortality rates are shown in Table 3. Most of the risk model variables indicated in the previous report [6] showed statistically significant relationships with higher crude operative mortality rates.

**Table 3:** Patients’ characteristics and crude operative mortality rates

Variables	Operative death (*n* = 173)		Alive (*n* = 6488)		*p* value
	No.	%	No.	%	
Age					< 0.001
≤ 59	17	9.8	1261	18.9	
60–64	24	13.9	1194	17.9	
65-69	20	11.6	1531	23.0	
70–74	42	24.3	1421	21.3	
75–79	47	27.2	875	13.1	
≥ 80	23	13.3	379	5.7	
Male	158	91.3	5592	84.0	0.007
Preoperative ADL (any assistance)	13	7.5	147	2.2	< 0.001
COPD	21	12.1	450	6.8	0.004
Weight loss > 10%	36	20.8	516	7.7	< 0.001
Cancer metastasis/relapse	3	1.7	59	0.9	0.197^a^
Platelet < 120,000/μl	10	5.8	221	3.3	0.067
Albumin < 3.5 g/dl	62	35.8	911	13.7	< 0.001
BUN < 8 mg/dl	4	2.3	140	2.1	0.785^a^
Serum Na < 138 mEq/l	33	19.1	566	8.5	< 0.001
PT-INR > 1.25	6	3.5	122	1.8	0.136^a^
WBC < 4500/μl	10	5.8	509	7.6	0.350

*ADL* activity of daily life, *COPD* chronic obstructive pulmonary disorder, *BUN* blood urea nitrgen, *PT-INR* prothrombin time-international normalized ratio, *WBC* white blood cell

^a^Fisher’s exact test

The response distribution in each QI and relationship between each result of QIs and the crude operative mortality rates are shown in Table 2. Three-hundred and eighty-nine (45.9%) out of 847 institutions certified by JSGS as of 2014 and 98 (86.7%) out of 113 institutions certified by JES as of 2014 responded to the questionnaire. In Qs1-6, the institutions which were accredited by or related to JSS (Q1) or certified by JES (Q3) and those with board-certified esophageal surgeon or esophagologist by JES (Q5 or Q6, respectively) showed significantly lower crude operative mortality rates. In 454 departments that responded to the questionnaire, the numbers of group B that answered “performed in principle” were 325 (71.6%), 178 (39.2%), 330 (72.7%) and 308 (67.8%) in QIs 7, 8, 9 and 10, respectively. Group B showed a significantly lower crude operative mortality in Q9 and Q10, while group C (performed at doctor’s discretion) resulted in worse results in Q7 and Q8. Q11 was excluded from the analysis because of its low recommendation grade in the guidelines [15].

### Relationship between the medical care system in relation to employment of board-certified surgeons at the department and the risk-adjusted odds ratio for operative mortality (AOR)

**Fig. 2:**
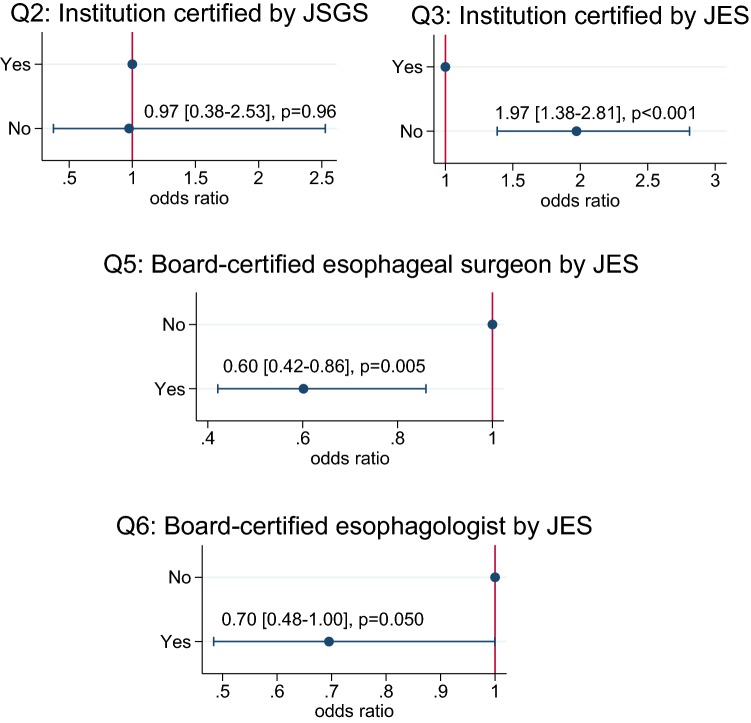
Relationship between board certification systems of surgeons and institution and the AOR in esophagectomy and reconstruction in Japan. The results show point estimates of odds ratio and 95% confidence intervals. *JSGS* Japanese Society of Gastroenterological Surgery, *JES* Japan Esophageal Society

The results after risk-adjustment using the factors mentioned in Materials and Methods are shown in Fig. 2. The numbers of the patients who showed operative death in the departments not accredited by or related to JSS and those in the departments without board-certified surgeons by JSGS were only 13 (0.2%) and 52 (0.8%), respectively. Therefore, neither Q1 nor Q4 proceeded to multivariable analyses.

1. Q2: There was no difference in the AOR between departments certified by JSGS (*n* = 389) and departments that were not (*n* = 65).
2. Q3, Q5 and Q6: The AOR was significantly lower in departments certified as training sites for esophageal surgeons by JES (*n* = 98) than in non-certified departments (*n* = 356) (*p* < 0.001). The AOR was significantly lower in departments with board-certified esophageal surgeons (*n* = 95) than in those without board-certified esophageal surgeons (*n* = 359) (*p* = 0.005). Similarly, there was a strong tendency that it was lower in departments with board-certified esophagologists (*n* = 159), which are a prerequisite for application for board-certified esophageal surgeons, than in those without (*n* = 295) (*p* = 0.050).

### Relationship between the rate of implementation of various QIs and the AOR

**Fig. 3:**
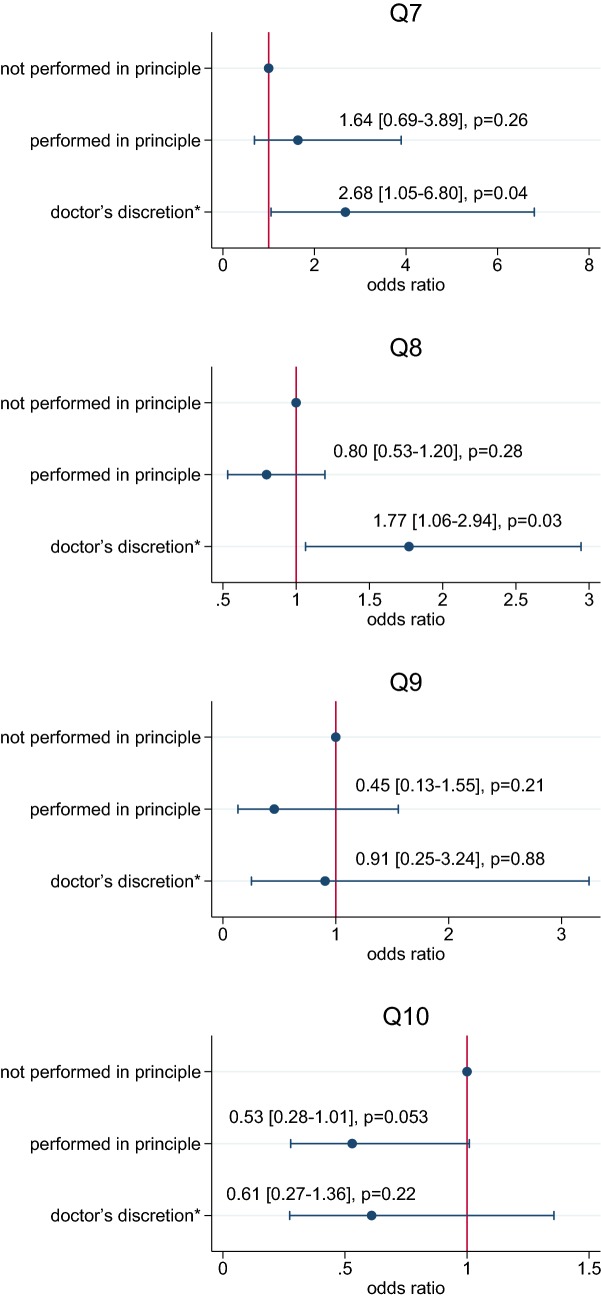
Relationship between process indicators related to esophagectomy and reconstruction and the AOR in Japan. The results show point estimates of odds ratio and 95% confidence intervals. Asterisk doctor’s discretion indicates “recommended by the institution, but performed at the doctor’s discretion”

Relationship between the rate of implementation of various QIs and the AOR is shown in Fig. 3. Q11 was not included because of its low level of recommendation in the guidelines.

1. *Q7: Screening for synchronous head and neck cancer in new patients with esophageal cancer.*
  Groups A, B and C had 39, 325 and 90 departments, respectively, and most departments performed the screening. There was no difference of the AOR between groups A and B, and the outcome was rather better in group A than in group C (*p* = 0.04), suggesting that doctor’s discretion might result in worse outcome as compared to no implementation of this QI.
2. *Q8: Perioperative steroid administration in esophagectomy and reconstruction*.
  Groups A, B and C had 215, 178 and 61 departments, respectively, showing that while some departments administered steroids in the perioperative period, others did not. There was no difference in the AOR between groups A and B, and the outcome was rather better in group A than in group C (*p* = 0.03), suggesting the same condition as Q7 mentioned above.
3. *Q9: Lymph node dissection around the bilateral recurrent laryngeal nerves*.
  Groups A, B and C had 22, 330 and 102 departments, respectively, revealing that systematic lymph node dissection has been widely used. There was no difference of the AOR between groups A, B and C.
4. *Q10: Neoadjuvant chemotherapy for resectable stage II/III thoracic esophageal cancer.*
  This QI is exclusively set on the basis of a randomized clinical trial performed in Japan [15]. Groups A, B and C had 53, 308 and 93 departments, respectively, indicating that many departments selected the standard of care. There was a strong tendency that the AOR of group B was better than that of group A (*p* = 0.053).

## Discussion

At present, 7 years have passed since the start of registration of surgical cases in the NCD, and the NCD has grown to a large-scale database that could potentially fully represent the reality of surgery throughout Japan [4, 5]. The JSGS has actively conducted research in various fields using these data [6–13, 18]. In the future, it would be desirable to attempt to evaluate the quality of medical care on a nationwide scale using structure indicator benchmarks, such as the institution system, regarding the provision of medical services, and process indicator benchmarks, such as the status of implementation of the standard of care. This study was planned and conducted as a pilot project for the above objectives for esophageal, gastric, colorectal, liver, biliary tract, pancreatic, lung and breast cancer, using data obtained from a questionnaire survey of departments registered in the NCD in 2014 and surgical case data in 2013 and 2014 from the departments that responded to the questionnaire. As the first report, this paper reports the actual status of surgical treatment of esophageal cancer in Japan, the significance of board certification of surgeons and departments, and 5 process indicators related to esophagectomy and reconstruction.

### Significance of board certification of surgeons and institutions

Konno et al. [19] reported that participation of gastroenterological surgeons who were board certified by the JSGS contributed to favorable operative mortality after eight gastroenterological procedures mentioned before in Japan and that the number of board-certified surgeons in gastroenterological surgery per hospital could be a surrogate marker of operative mortality. We examined the relationship of the board certification of surgeons and institutions related to esophagectomy and reconstruction by the JSS, JSGS and JES to the operative mortality as an indicator of the level of medical care. The relationship concerning JSS could not be accurately assessed, because most of the departments that responded to the questionnaire belonged to institutions that were accredited by or related to JSS. However, the results suggested that the board certification of institutions as training sites for esophageal surgeons by JES and the presence of board-certified esophageal surgeons significantly reduced the AOR (Fig. 2). Furthermore, there was a strong tendency that the presence of board-certified esophagologists by JES, which is a prerequisite for the board-certified esophageal surgeons, showed a better AOR (Fig. 1). The NCD, JSGS and JES have begun to jointly investigate the significance of board-certified esophageal surgeons using larger-scale NCD data.

### Five process indicators related to esophagectomy and reconstruction

General clinicians have not been fully aware of the significance of evaluation of the quality of medical care based on the measurement of a small number of QIs in clinical settings. This questionnaire survey was conducted to improve their understanding.

Because the QIs related to the surgical treatment of esophageal cancer used in this study did not necessarily have high grades of recommendation according to the 2012 Guidelines for Diagnosis and Treatment of Carcinoma of the Esophagus [15], which was the latest guideline when the survey was conducted in 2014, the measurement of these QIs may have a limited significance. Ideally only those QIs should have been selected whose implementation would be an indicator of a high quality of medical care. However, there are not many health services that can be highly recommended based on evidence not only at the time of the questionnaire survey, but even in the current surgical treatment of esophageal cancer (the 2017 Guidelines for Diagnosis and Treatment of Carcinoma of the Esophagus [20, 21]). Therefore, for the present study, a discussion was held among the council members of the JES to select 5 QIs that would be useful to enlighten general clinicians about the significance of QI measurement.

This study revealed that a considerable part of departments executed clinical practices recommended by the guidelines in Japan (Table 2). On the other hand, it was somewhat surprising that the AOR rates in departments in which doctor’s discretion was permitted might be rather worse than in those which did not perform the QIs 7 and 8 in principle. This fact might indicate that, in the field of esophageal surgery like an esophagectomy, establishing and nationwide standardization of high levels of evidences would yield much improvement of a quality of medical care. Contrarily, regarding Q10 which is based on one of the best evidences in Japan even at present [15], there was a strong tendency that group B showed the lower operative mortality rates as compared to group A, suggesting that departments performing a standard therapy might achieve better short-term outcomes. In the future, it needs to be evaluated whether an improvement in the rate of implementation over time through significant QI selection and feedback of results actually leads to an eventual improvement in the level of medical care including a quality of life and ADL [22, 23].

### Limitations of this study

The first limitation of this study was that it was based on a questionnaire survey. Since any users who had been registered in the NCD as a user and obtained an ID could answer the questionnaire, the questionnaire data may not necessarily reflect the opinions of the departments. The second limitation is that the questionnaire response rate was 49.6%, suggesting that the results do not fully reflect the actual situation in the entire country. However, considering the fact that 95% of esophagectomy and reconstruction cases throughout Japan are registered in the NCD [5] and that most of the departments that responded to the questionnaire were accredited by or related to the JSS and nearly 87% of institutions responded were certified by JES (98 out of 113 as of 2014), it is possible that the results reflected the actual status of surgical treatment of esophageal cancer throughout Japan to some extent. In this questionnaire survey, the response rate of high volume centers tended to be higher than that of low volume centers (data not shown), suggesting a possibility that the effect of each QI might be underestimated. The third limitation was that, as described above, the grounds for selecting the QIs were not clear. In the future, QIs need to be selected using a method like the Delphi method and their appropriateness evaluated [24].

## Conclusions

In this study, we investigated the relationship of the medical care system in relation to esophageal surgery and the status of implementation of the selected QIs to the operative mortality in each department, through conducting a questionnaire survey of the departments registered in the NCD. The results suggested that the board certification of institutions as training sites for esophageal surgeons and the presence of board-certified esophageal surgeons at departments performing the treatment are associated with reduced operative mortality rates. Through activities like this, it is desirable to raise the awareness of the significance of objective evaluation of the quality of surgical treatment for esophageal cancer in clinical settings. In the future, we propose to report the results of questionnaire surveys conducted to evaluate the status of management of also other gastrointestinal cancers (gastric, colorectal, pancreatic, liver and biliary tract cancer).

## Abbreviations

NCD: National Clinical Database
AOR: Risk-adjusted odds ratio for operative mortality
JSS: Japan Surgical Society
JSGS: Japanese Society of Gastroenterological Surgery
JES: Japan Esophageal Society
QI: Quality indicator
